# The use of carbon monoxide breath test to detect the effect of iron overload on erythrocyte lifespan in MDS

**DOI:** 10.3389/fonc.2022.1058482

**Published:** 2022-11-29

**Authors:** Yao Zhang, Yan Qian, Lu-xi Song, Chao Xiao, Chun-kang Chang

**Affiliations:** Department of Hematology, Shanghai Sixth People’s Hospital Affiliated to Shanghai Jiao Tong University School of Medicine, Shanghai, China

**Keywords:** MDS, erythrocyte lifespan, EPO, cytokines, iron removal therapy

## Abstract

**Objective:**

To investigate the effect of iron overload (IO) on red blood cell (RBC) lifespan in MDS patients with the use of carbon monoxide breath test

**Methods:**

The red blood cell lifespan of 93 patients with myelodysplastic syndrome (MDS) and 22 healthy volunteers in the control group were measured by alveolar gas carbon monoxide (CO) assay, with the detection of liver iron concentration, iron metabolism index, erythropoietin (EPO) concentration, peripheral blood inflammatory cytokines, etc. The MDS patients were divided into the severe IO group, mild IO group and non IO group according to liver iron concentration. The effect of IO on RBC lifespan was analyzed in MDS patients.

**Results:**

The RBC lifespan of MDS patients in the severe IO group was significantly lower than that in the mild IO group (p<0.05), while the RBC life span in the mild IO group was significantly lower than that in the non IO group (p<0.05). The expression of inflammatory cytokines in the severe IO group was significantly higher than that of the mild and non IO groups. After receiving iron removal treatment(ICT), the expression of inflammatory cytokines was decreased significantly, and the RBC lifespan was significantly prolonged (p<0.05).Besides, liver iron concentration was significantly positively correlated with EPO concentration, while EPO concentration was significantly negatively correlated with RBC lifespan, especially in the MDS-RS subgroup. The RBC lifespan in the EPO>1000 group was significantly lower than that in the EPO<1000 group.

**Conclusion:**

IO can shorten RBC lifespan in MDS patients, which may be result from the increase of endogenous EPO and the over-expression of inflammatory cytokines. After ICT, the ineffective hematopoiesis caused by increased EPO may reduced and the decrease of inflammatory cytokine may significantly prolong the RBC lifespan in MDS patients.

## Introduction

Myelodysplastic syndromes (MDS) patients may suffer from iron overload as a result of a longterm transfusion of red blood cells (RBC) and/or combined with abnormal iron metabolism. Most of the causes of anemia in MDS patients are related to ineffective hematopoiesis caused by morbid hematopoiesis. In the process of division and maturation, young RBCs are destroyed or die before they mature and enter the peripheral circulation for some reasons. There is an obvious contradiction between the decrease of peripheral blood cells and the excessive proliferation and apoptosis of bone marrow cells in MDS patients. The increased level of apoptosis may be the main reason for the ineffective hematopoiesis of bone marrow in MDS patients (1). The pathological phenotype of low-risk MDS patients, especially MDS-RA and RARs sub-types, is mainly limited to erythroid cell injury, and their erythroid cell apoptosis begins in the early stage of stem cells. In contrast, the percentage of apoptotic cells in high-risk MDS patients (with too many primordial cells) is lower than that in normal subjects, which is similar to the percentage observed in acute leukemia (2).

In recent years, a new technology of rapid and simple CO breath test for the determination of RBC life span is being applied in clinic (3). The lifespan of RBCs in circulation reflects the quality or intrinsic defect of mature RBC. Under physiological conditions, the production and destruction of RBCs is in dynamic balance. The average life span of RBC in healthy adults is 115 (70 ~ 140) days. In the domestic consensus, while the average life span of RBC in normal people with CO breath test method is 126d, and the 95% confidence interval is 75-177d (4). After excluding hemolysis caused by other reasons, such as autoimmune hemolysis, drug-related hemolysis, PNH, congenital spherocytosis, G-6-PD deficiency, hemoglobinopathy, etc., the lifespan of RBC can be used to evaluate the degree of excessive apoptosis or destruction of RBC.

Erythrocytes which contain neither nucleus nor mitochondria, undergo apoptosis mainly by the increase of cytoplasmic Ca^2+^ concentration, the increase of reactive oxygen species(ROS), the formation of ceramide and the induction of protein phosphorylation. With the stimulation of ROS, RBCs promote the synthesis and release of platelet activating factor(PAF), then activate and stimulate sphingomyelinases, which lead to the production of ceramide. Further, ceramide activates intracellular invertase, catalyzes the translocation of phosphatidylserine (PS) existing in the inner membrane to the outer membrane, exposes phosphatidylserine, and induces erythrocyte apoptosis (5). Iron overload leads to the production of intracellular free radicals, especially in MDS-RA and RARs subtypes, which leads to oxidative damage and induces apoptosis of hematopoietic progenitor cells (6). *In vitro*, excessive iron load promotes apoptosis of immature erythrocyte through increased intracellular ROS generation. Erythroid burst forming unit colony and differentiation into mature erythrocytes were significantly inhibited. Iron removal treatment(ICT) can reduce the level of ROS in cells, inhibit apoptosis, and restore the differentiation to mature RBCs (7).

Does IO shorten the life span of RBCs in MDS patient? Have been an improvement in the lifespan of RBC after ICT? Does the high expression of EPO *in vivo* influence the lifespan of RBC in MDS patients? Do the increased number of bone marrow blasts and the expression of inflammatory cytokines affect the life span of RBC? This study hopes to answer these questions.

## Materials and methods

This study was conducted in accordance with the Declaration of Helsinki and was approved by the Ethics Committee of our hospital [No.2022-KY-171(K)]. Written informed consent was obtained from all participants.

### Cases and groupings

We retrospectively identified and assessed patients with a consecutive series of MDS from July 2021 to October 2022, and excluded cases who were combined with hemolysis caused by other reasons, such as autoimmune hemolysis, PNH, congenital spherocytosis, G-6-PD deficiency, hemoglobinopathy, etc. After exclusion checks, 93 patients with MDS, 22 healthy volunteers were included in this study. MDS diagnosis was performed in accordance with the 2019 expert consensus (8).Typing was in accordance with the 2016 WHO classification standards (9). IPSS-R scores were used for evaluation and grading (10). The liver iron content in patients with MDS was evaluated by energy spectrum CT. Based on the relevant literature, we defined liver virtual iron content (LVIC) < 1.34(mg/ml) as the normal group, 1.34 ≤ LVIC < 1.85 (mg/ml) as the mild IO group, 1.85 ≤ LVIC < 2.69 (mg/ml) as the moderate IO group, 2.69 ≤ LVIC < 4.03 (mg/ml) as the severe IO group, and 4.03 (mg/ml) ≤ VIC as the extremely severe IO group (11). According to the LVIC, we divided patients with MDS into severe IO group(LVIC classification: severe and extremely severe IO groups), mild IO group(LVIC classification:mild and moderate IO groups)and non IO group(normal group). Healthy volunteers were included into the control group. The clinical characteristics of the patients are shown in **Table 1**.

**Table 1:** General conditions of patients in MDS and control group.

	IO group (n=55)		Non IO group (n=38)	Control group (n=22)	*P* value
	severe IO group (n=32)	Mild IO group (n=23)			
**Age (m) (range)**	53 (25~90)	56 (32~81)	52 (32~83)	55 (32~83)	>0.05
**Gender (M/F)**	15/17	11/12	18/20	10/12	>0.05
**WHO classification,n (%)**					
MDS-SLD	0 (0%)	1 (4.3%)	0 (0%)	–	
MDS-MLD	15 (46.9%)	7 (30.4%)	13 (34.2%)	–	
MDS-RS	9 (28.1%)	3 (13.0%)	0 (0%)	–	
MDS-EB	6 (18.8%)	8 (34.8%)	20 (52.6%)	–	
tAML	2 (6.3%)	3 (13.0%)	5 (13.2%)	–	
5q-	0 (0%)	1 (4.3%)	0 (0%)	–	
**IPSS-R risk,n (%)**					
Very low	7 (21.9%)	2 (8.7%)	4 (10.5%)		
Low	16 (50.0%)	8 (34.8%)	12 (31.6%)		
Intermediate	2 (6.3%)	5 (21.7%)	7 (18.4%)		
High	5 (15.6%)	6 (26.1%)	9 (23.7%)		
Very high	2 (6.3%)	2 (8.7%)	6 (15.8%)		
**Anemia,n (%)**	32 (100%)	21 (91.3%)	25 (65.8%)	0 (0%)	<0.05
mild	0 (0%)	4 (19.0%)	9 (36.0%)	0 (0%)	
moderate	12 (37.5%)	9 (42.9%)	11 (44.0%)	0 (0%)	
severe	20 (62.5%)	8 (38.1%)	5 (20.0%)	0 (0%)	
**TD,n (%)**	30 (93.8%)	5 (21.7%)	0 (0%)	0 (0%)	<0.05
**EPO (mIU/ml)**	(1934~24935)	(262.9~5132)	(19.4~746)	(12~75)	<0.05
**ASF (ng/ml)**	(1867~21300)	(895~3265)	(49~1621)	(30~168)	<0.05
**TS (%)**	(86~99)	(72~85)	(55~71)	(42~53)	<0.05
**BMF**	4/32	1/23	0/38	0/22	<0.05

MDS, myelodysplastic syndrome; WHO, World Health Organization. IPSS-R, Revised International prognostic scoring system. The risk classification is determined according to the numerical rating system at the time of initial diagnosis. MDS-SLD, single lineage dysplasia; MDS-MLD, multi lineage dysplasia; MDS-RS, ring sideroblasts; MDS-EB, excess blasts; tAML, MDS transformed acute myeloid leukemia; TD, Transfusion dependence; Transfusion-dependent: at least 4U of RBC with 8 weeks for hemoglobin < 6 g/dL. ASF, adjusted serum ferritin; TS, transferrin saturation. BMF, Bone marrow fibrosis; Anemia classification: male 90≤Hb<130g/l, female 90≤Hb<110g/l is mild anemia, 60≤Hb<90g/l is moderate anemia, and Hb<60g/l is severe anemia.

### Treatment methods

The patients with MDS in the low-risk group had taken the drugs of cyclosporine, thalidomide and other immunomodulatory in combination with the hematopoiesis promoting treatments. The patients with MDS in the medium and high-risk group were treated with demethylation (azacytidine or decitabine), while tAML patients were treated with demethylation in combination with Bcl-2 inhibitor (venecla) or allogeneic hematopoietic stem cell transplantation(allo-HSCT). 72 patients with MDS had a history of RBC transfusion, among which 35 patients were complicated with RBC transfusion dependence(TD). The median volume of RBC transfusion in the past year was 16 (4~62) U. Among them, 28 cases who had an expected survival of ≥ 1 year, with the total RBC transfusion of more than 80 U (1 u RBC for red blood cells isolated from 200 ml whole blood), the test of adjusted ferritin (ASF) ≥ 1000 ng/ml and the RBC transfusion dependence received the treatment of iron chelation therapy (ICT) (12): desferriamine mesylate 20-60 mg • kg^-1^ • d^-1^ is continuously pumped subcutaneously for 12 hours or desilaros 30 mg • kg^-1^ • d^-1^ is taken orally, for 14 days as a course (13). The lifespan of RBC was measured in 16 patients before and after ICT. All patients had more than 4 courses of ICT, of which 12 cases completed 8 courses or more, and 7 cases completed 10 courses or more. There was no history of RBC infusion in healthy volunteers, with ASF <500 ng/ml when RBC lifespan was tested. All MDS patients in this study did not receive mitoxantrone and platinum recently.

### RBC lifespan measurement

**Figure 1:**
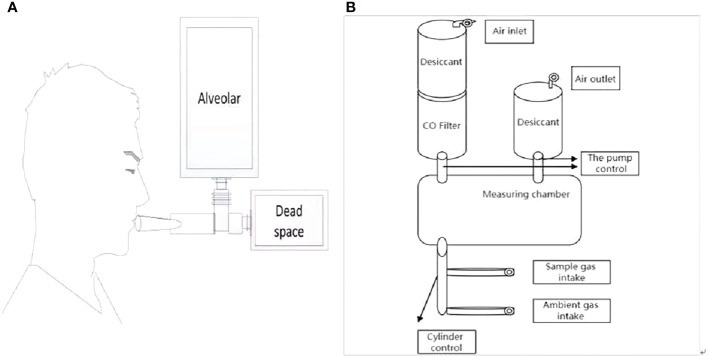
Experimental set up. **(A)** Breath sample collecting system. **(B)** Overview of measurement instruments structure.

We evaluated alveolar endogenous CO concentration by automated instrument(i.e. ELS TESTER, model RBCS-01, Seekya Biotec Co. Ltd, Shenzhen, China) with the used of non-dispersive infrared spectroscopy, and collected the paired samples of room air and alveolar gas for the RBC lifespan measurements conducted by Levitt’s formula (4). The samples of alveolar gas were collected in the morning (8.00– 11.30) without fasting requirement, while smoking patients were asked to quit smoking for 24 hours. After a deep inspiration, each participant held his or her breath for 10’s and then exhaled into a collection system using a mouth-piece (**Figure 1A**). The collection system discarded the first 300 ml of dead space gas and then directed subsequent alveolar air automatically into a foil collection bag. If needed, the procedure was repeated until the collected air sample reached the collection bags capacity of 1000 ml. The filled bag was detached and sealed. Atmospheric samples were collected just after breath sampling. Alveolar gas and atmospheric samples were stored at room temperature and analyzed within 5 days. On the same day as alveolar gas sampling, peripheral vein blood samples were collected for routine hemoglobin measurement.The working flow chart of gas circuit of measuring instrument is illustrated in **Figure 1B**. The instrument operator connected the paired air-alveolar gas samples to inlets, entered the participants blood hemoglobin concentration data, and then pressed the start button, triggering the instrument to complete a series of automated measurements encompassing the following steps. First, the quality of a collected alveolar sample was checked by measuring its CO2 concentration. Only alveolar gas samples containing <5% CO2 were considered to be diluted. Second, in order to eliminate molecules that might interfere with the infrared detection of CO, the sample gas was advanced into a de-interference system with an absorbent mixture consisting mainly of soda asbestos. The absorbed interference molecules were mainly H2O and CO2. Third, a paired measurement technique was employed to determine the CO concentration difference between alveolar gas and atmospheric air. Fourth, any endogenous alveolar samples found to be diluted (i.e. having <5% CO2) were normalized to a 5% CO2 status. Any endogenous alveolar sample in which dilution was found (i.e., CO2 content <5%) was normalized to the state of 5% CO2. Finally, the lifespan of RBC was calculated by Levitt’s formula (14) based on the parameters of the pre-imputed hemoglobin concentration and corrected (if necessary) endogenous alveolar CO concentration. The instrument reports the following data for the subject: alveolar CO2, endogenous alveolar CO, and RBC lifespan.

### Detection of iron metabolism indexes and EPO

Serum ferritin (SF) and erythropoietin (EPO) were determined by radioimmunoassay. Serum iron and total iron binding capacity were detected by ferrous nitrogen colorimetry. C-reactive protein (CRP) was detected by immunoturbidimetry. When CRP > 10 mg/l, ASF = sf/log 10 CRP and CRP ≤ 10 mg/l, ASF = SF (15), the data of SF and CRP was collected on the same day.

### Detection of liver iron concentration by Energy spectrum CT

The same 2 × 192-layer DECT scanner (Siemens SOMATOM Force) was used for all examinations. The detection protocol used dual energy (De, 100 and 150 kvp) with a thin filter to improve the separation of the two spectra. The image data was processed with the software prototype (DE IronVNC; syngo.via Frontier; Siemens Healthineers). The liver map was made by decomposing the basic materials into air, water, and iron. Skilled CT diagnosis radiologists performed the image analysis. The VIC of the liver was measured by placing three large freehand regions of interest(ROIs) at the hepatic vault and about 1 cm below the hepatic capsule, excluding the larger vessels near the portal vein and inferior vena cava. Moreover, ROIs included the left lobe and the anterior part of the right lobe.

### Detection of peripheral blood cytokine

Interleukin(IL)-6, IL-10, Tumor necrosis factor (TNF)-α, Interferon (IFN)-y, IL-17A,IL-1β, IL-12p70, IFN-a, IL-8, IL-2, IL-4 and IL-5 in peripheral blood were detected by ELISA. The detection was carried out in strict accordance with the product instructions. None of the patients had infectious fever at the time of sampling.

### Statistical analysis

SPSS 19.0 was used for statistical analysis. The normal distribution data was expressed as “mean ± standard deviation”. The independent sample t-test was employed for the comparison of the two groups of data, and the paired t-test was used for the data before and after ICT.The data with non normal distribution was expressed in the median. Mann Whitney U test was used for the comparison between the two groups. Wilcoxon test was used for the comparison between the two groups before and after treatment, while Spearman analysis was applied for correlation analysis, in which *P* < 0.05 indicated that the difference was statistically significant.

## Result

**Figure 2:**
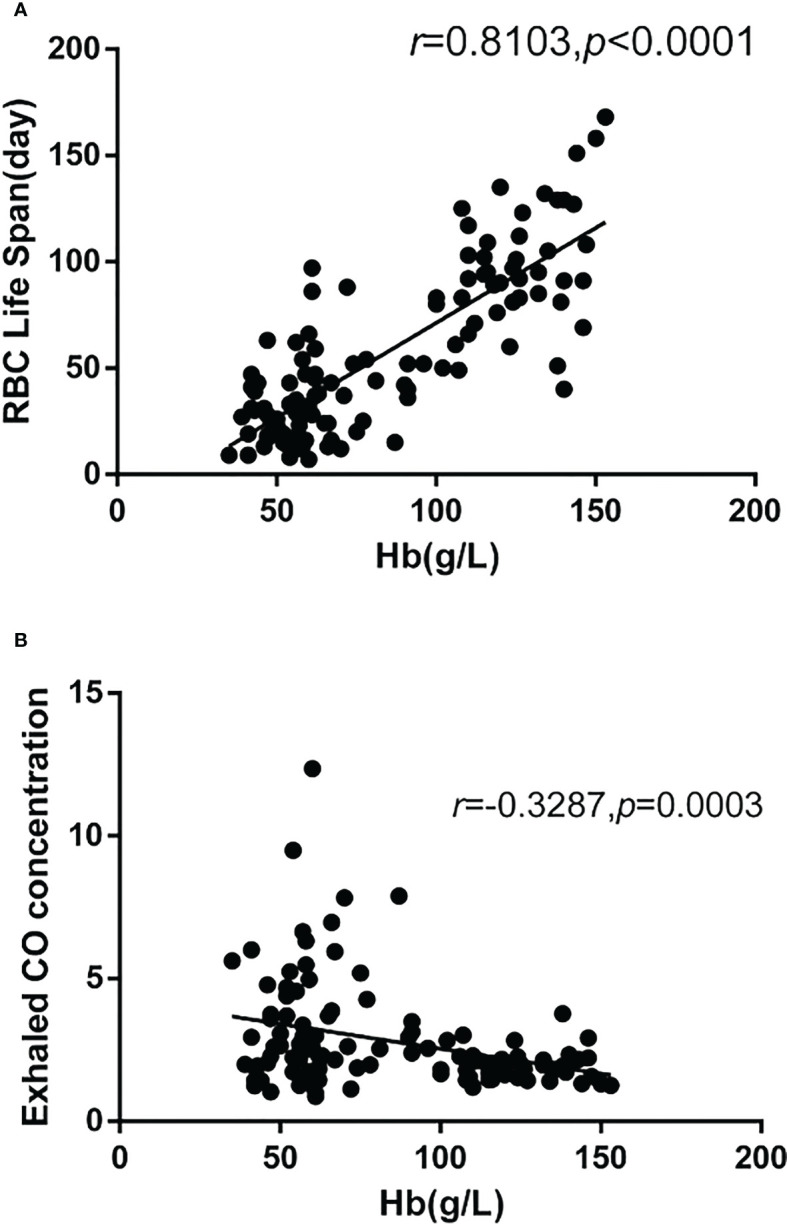
There were 115 cases in the MDS group and the control group. The correlation analysis results showed that: **(A)** The hemoglobin was significantly positively correlated with the RBC life span(r=0.8103, p<0.0001), **(B)** The hemoglobin was significantly negatively correlated with the concentration of CO (r=-0.3287, p=0.0003).

**Figure 3:**
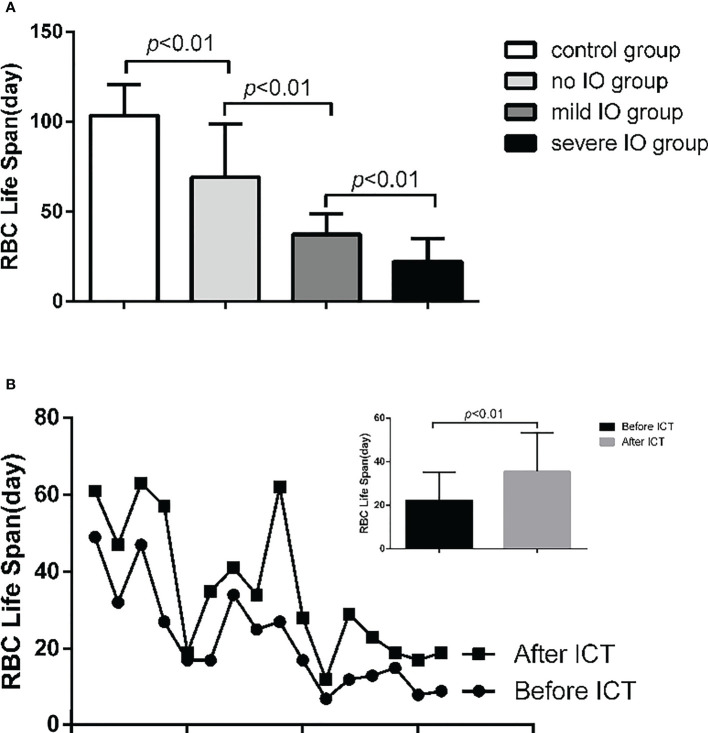
**(A) **The RBC life span of mild IO group was significantly lower than that in non IO group (p<0.01); and the severe IO group was significantly lower than that of the mild IO group (p<0.01); and the non 10 group was significantly lower than that of the control group (p<0.01). **(B) **16 MDS patients in IO group were tested for RBC life span before and after ICT. The results showed that the RBC life span in 10 group increased significantly after ICT (p<0.01).

**Figure 4:**
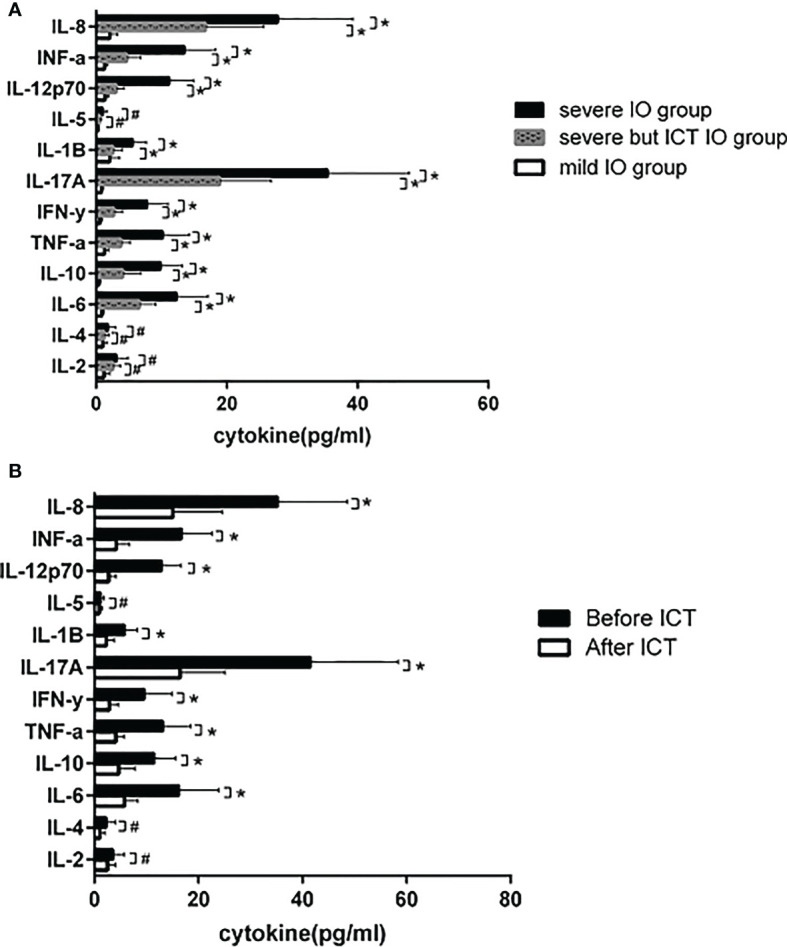
**(A)** 15 MDS patients with severe IO, 10 MDS patients with severe IO but after ICT, and 32 MDS patients with mild IO received peripheral blood cytokine detection. IL-6 (12.3 ± 4.8 vs 0.9±0.3), IL-10 (9.9 ± 3.3 vs 0.4 ± 0.3), TNF-α (10.2±4.1 vs 1.3±0.6). IFN-y (7.8±3.2 vs 0.6±0.3), IL-17A (35.4±12.5 vs 0.8±0.3), IL-1β (5.6 ± 2.1 vs 2.1±1.5), IL-12p70 (11.2 ± 3.7 vs 1.4±0.5), IFN-a (13.5±4.8 vs 1.3±0.5) and IL-8 (27.8 ± 11.5 vs 2.1 ± 1.2) in severe IO group were significantly higher than those in mild IO group (*p<0.05), while in severe IO group but with regular ICT, IL-6 (12.3±4.8 vs 6.6±2.5), IL-10 (9.9±3.3 vs 4.1±2.8), TNF-α (10.2±4.1 vs 3.8±1.4). IFN-y (7.8±3.2 vs 2.7±1.4), IL-17A (35.4±12.5 vs 18.9±7.9), IL-1β (5.6±2.1 vs 2.6±1.4), IL-12p70 (11.2±3.7 vs 3.1±1.2), IFN-a (13.5±4.8 vs 4.742.1) and IL-8 (27.8±11.5 vs 16.748.9) were significantly lower than those in severe 10 patients without ICT (*p<0.05); However, there was no significant difference in the expression of IL-2, IL-4 and IL-5 (#*p*>0.05). **(B)** 12 MDS patients with severe IO tested those cytokines before and after ICT. After ICT, IL-6 (16.5±7.6 vs 5.8 ± 2.6), IL-10 (11.5±4.3 vs 4.7±3.1), TNF-α (13.2±5.4 vs 4.1±1.7). IFN-y (9.6±5.3 vs 2.9±1.7), IL-17A (41.6±16.8 vs 16.5±8.6), IL-1B The expression of cytokines such as (5.8±2.5 vs 2.3±1.6), IL-12p70 (12.9±3.8 vs 2.8±1.4), IFN-a (16.8±5.7 vs 4.2±2.6) and IL-8 (35.2±13.5 vs 15.1 ± 9.6) was significantly lower than before (*p<0.05), and there was no significant difference in the expression of IL-2, IL-4 and IL-5 (#*p*>0.05).

**Figure 5:**
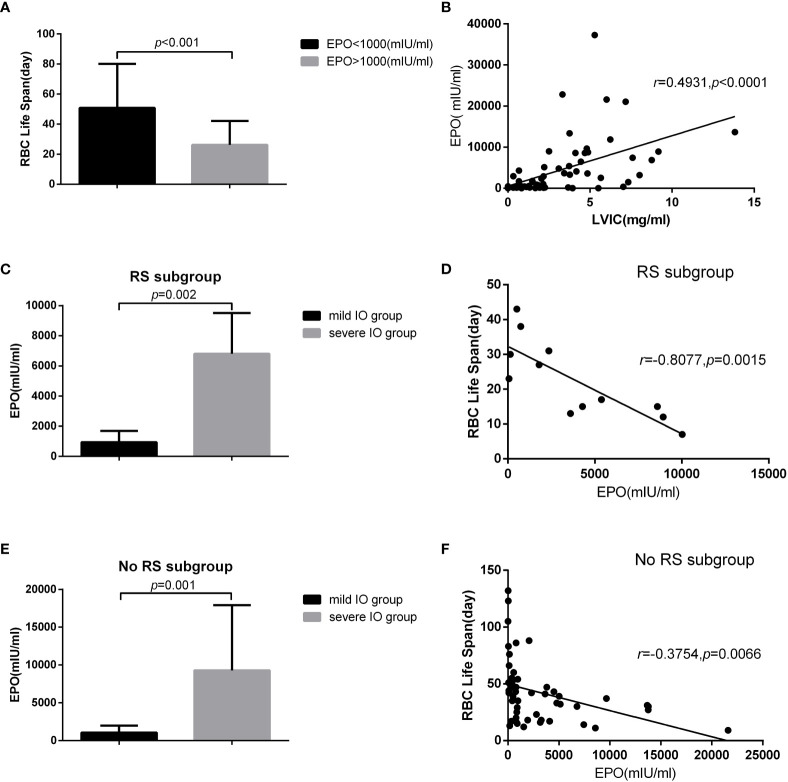
27 MDS patients in non IO group and 36 MDS patients in IO group who were not use EPO within one month were included in the analysis. There were 12 cases in MDS-RS and 51 cases in non MDS-RS subgroup. The results showed that: **(A)** the RBC life span in EPO>1000mlU /ml was significantly lower than that of EPO<1000mlU/ml (50.81±29.29 vs 26.23±15.87, p<0.001). **(B)** There was a significant positive correlation between liver VIC and EPO concentration (*r*=0.4931, p<0.001). In MDS-RS subgroup, **(C)** EPO concentration in severe IO group was significantly higher than that in mild IO group (6802.50 ± 2709.02 vs 934.22 ± 763.49, p<0.001), **(D)** and EPO concentration was significantly negatively correlated with RBC life span (r=-0.8077, p<0.01). In the non MDS-RS subgroup, **(E)** EPO concentration in severe 10 group was also significantly higher than that in the mild IO group (9290.09±8638.86 vs 1050.10 ± 930.81, p<0.001), **(F)** and EPO concentration was significantly negatively correlated with RBC life span (r=-0.3754, p<0.01).

**Figure 6:**
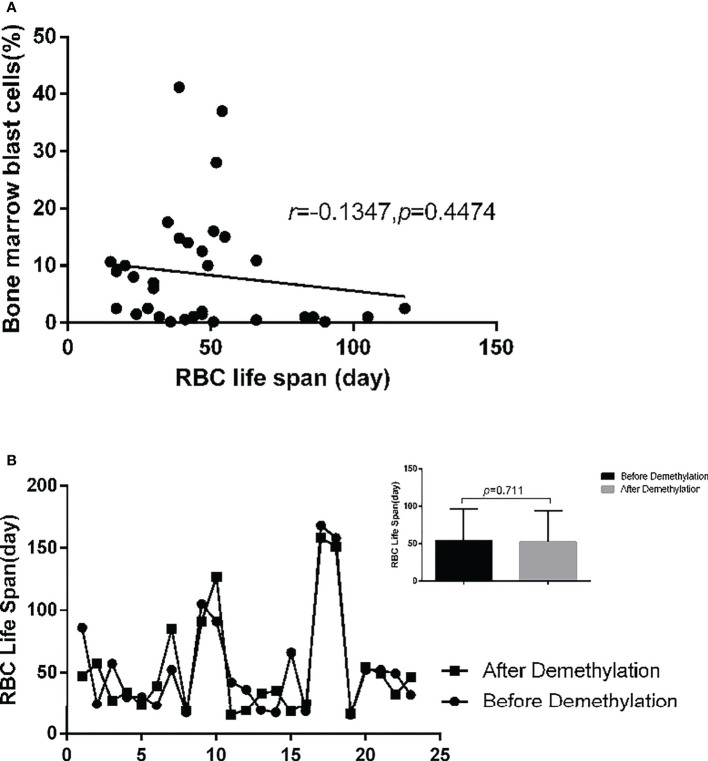
34 high-risk MDS and tAML patients(except 6 patients who underwent allogeneic stem cell transplantation) were included in the analysis. **(A)** There was no significant correlation between the number of bone marrow primordial cells and RBC life span (*r*=-0.1347, *p*=0.4474). **(B)** 23 patients with medium and high-risk MDS patients were detected RBC life span before and after demethylation drug treatment. It shows that demethylation treatment has no significant effect on the RBC life span (*p*=0.711).

In the MDS group, 83.9% of patients develop anemia and 87.1% of patients suffer from the lifespan shortening of RBC, while in the control group, none of patients develop anemia and the lifespan of RBC was all within the normal range(>75d). The correlations between hemoglobin and CO concentration, hemoglobin and RBC life span are shown in **Figure 2**. The effect of iron overload on the life span of RBC and the changes of RBC life span after ICT in MDS patients are shown in **Figure 3**. The expression of inflammatory cytokines in peripheral blood and the changes after ICT in MDS patients are shown in **Figure 4**. The correlation between EPO concentration and RBC lifespan and the differences of RBC lifespan in the EPO<1000 and EPO >1000 mIU/ml group among different MDS subgroups are shown in **Figure 5**. The correlation between the number of bone marrow primordial cells and the lifespan of RBC in the high-risk group MDS patients, and the effect of demethylation treatment on the lifespan of RBC in the high-risk group MDS patients are shown in **Figure 6**. In 5 tAML patients, the lifespan of RBC has been decreased during the treatment of demethylated and Bcl-2 inhibitor. However, after the recovery of hematopoietic, the lifespan of RBC has been increased. Since the number of cases is too small, the effect of Bcl-2 inhibitor on RBC life span cannot be determined.

## Discussion

70% of the endogenous CO in human body comes from the degradation of Hb after the destruction of RBCs, and the lung is the only way for the body to excrete CO. One mole of Hb is degraded to produce four moles of CO. The amount of Hb degradation in 24h can be calculated by measuring the endogenous CO concentration in alveolar gas. The formula that the total amount of Hb in the whole body is divided by the amount of Hb degradation in 24h is used to estimate the value of erythrocyte life in the detection period. The decomposition degree of RBCs in the body can be indirectly estimated by detecting the concentration of CO. Thus, the high concentration of CO can infer the high heme produced by the decomposition of RBCs. This study shows that the amount of hemoglobin in MDS patients is significantly negatively correlated with carbon monoxide (CO) concentration and is significantly positively correlated with the life span of RBC. The life span of RBC in MDS patients is significantly lower than that of the control group. For MDS patients, the shortening lifespan of RBC indicates the high rate of erythrocyte decomposition, which may be corresponds to the high degree of erythrocyte damage or apoptosis.

The life span of RBC in the severe IO MDS group is significantly lower than that in the mild IO MDS group and the non IO MDS group. After ICT, the lifespan of RBC in the IO patients are increased significantly. These results indicate that the IO can shorten the lifespan of RBC in MDS patients, which may be relevant to the aggravation of erythrocyte damage or apoptosis in patients.IO can cause the increase of reactive oxygen species (ROS) in the body. Previous studies have shown that the increase of reactive oxygen species production and oxidative stress in mouse models affects the expression of FoxO transcription factor, p38 mitogen activated protein kinase (MAPK), ataxia telangiectasia mutant (ATM) protein and other signal pathways. These changes are related to the loss of hematopoietic stem cells. Through these pathways, the signaling of reactive oxygen species may be involved in regulating the proliferation and apoptosis of normal and tumor hematopoietic progenitor cells (16). Previous studies in our center also shows that iron overload can promote erythroid cell apoptosis in patients with MDS by regulating HIF-1a/ROS signaling pathway (17), and promote mitochondrial breakage of mesenchymal stromal cells in patients with MDS by activating ampk/mff/drp1 pathway (18).

The reaction of erythroid system to tissue hypoxia is the pathophysiological basis of ineffective erythropoiesis. Tissue hypoxia increases the expression of erythropoietin (EPO), which induces the formation of new erythrocytes. In anemia cases related to ineffective erythropoiesis, the imbalance between red blood cell supply and demand still exists, in spite of tissue hypoxia and increased EPO. Therefore, patients with ineffective erythropoiesis are usually accompanied by too many young erythrocytes in their bone marrow (19). This study shows that the life span of RBC in MDS patients with EPO>1000 mIU/ml is significantly lower than that in MDS patients with EPO<1000 mIU/ml, which indicates that the life span of RBC in MDS patients with high EPO concentration decreases more seriously. This may be caused by that excessive concentration of endogenous EPO in MDS patients stimulates a large number of erythroid hyperplasia, and excessive ineffective hematopoiesis shortens the overall life expectancy of RBC. Moreover, there was a significant positive correlation between liver iron concentration and EPO. It suggests that iron overload affects the expression of endogenous EPO in MDS patients. Our previous research results show that heavy iron overload will promote the increase of endogenous EPO but affect the expression of EPO-stat5 pathway and inactivate EPO signal pathway (20). EPO concentration decreased after ICT treatment (21). It is speculated that the reason may be related to the increase of EPO production due to the feedback stimulus caused by blocked EPO signal.

The subgroup analysis shows that EPO concentration is significantly negatively correlated with RBC lifespan in both MDS-RS and MDS non RS groups. The expression of EPO in the severe IO group was significantly higher than that in the mild IO group. The correlation between EPO and RBC lifespan in the MDS-RS subgroup is significantly higher than that in the MDS non RS group. It indicates that Iron overload leads to the increase of endogenous EPO expression, and the increase of EPO leads to the increase of erythroblast and ineffective hematopoiesis, which is one of the reasons for shortening the life span of RBC in MDS patients.

In high-risk MDS patients, there is no significant correlation between the number of primordial cells and RBC lifespan. It shows that in MDS patients, bone marrow invasion of tumor cell leads to the function of impaired hematopoietic, which mainly affects the number of red blood cells but has no significant impact on RBC life.

Treatment related anemia has always been an unavoidable problem in the treatment of cancer patients. Lang et al. (22) shows that in addition to bone marrow inhibition, mitoxantrone and platinum in chemotherapy drugs can also promote the decline of red blood cells, resulting in obvious anemia and shortened red blood cell lifespan. This experiment shows that the treatment of demethylation only causes bone marrow suppression, but there is no significant difference in the lifespan of red blood cells before and after treatment. The change of erythrocyte lifespan is related to the remission of the disease. The treatment of demethylation has no significant effect on the lifespan of erythrocyte. However, the number of cases is small, and the effect of Bcl-2 inhibitor needs to be further clarified by increasing the number of cases.

Our study showes that the expressions of inflammatory cytokines IL-6, TNF-α, IFN-y, IL-17A, IL-1β, IL-12p70, IFN-a and IL-8 in peripheral blood of MDS IO group are significantly higher than those in the non IO group. It has been proved that iron overload could induced M1 polarization in RAW 264.7 macrophages, which is associated with the induction of ROS production in iron-treated cells with the characterization of elevated TNF (23). M1 macrophage polarization caused by Th1 cytokines, such as TNF and IFN-γ produce majorly proinflammatory cytokines, such as TNF, IL-1 α, IL-1 β, IL-6, IL-12, IL-23 and low levels of IL-10. The pro-inflammatory activity of these macrophages promote DNA damage by the release of intracellular free radicals (6, 24). Apoptosis triggered by bone marrow micro-environment and/or intrinsic cell is also regulated by different levels of cytokines. Inflammatory cytokines also reduce the sensitivity of EPO (25). ROS induces heme oxygenase-1 (HO-1) expression (26). HO-1 is an enzyme that catalyzes the degradation of heme and releases equimolar amounts of carbon monoxide (CO), biliverdin (BV) and Fe2 +. HO-1 and its products CO and BV are generally related to promote the antioxidant, anti-inflammatory and immunosuppressive activities of macrophages (27). The inhibition of HO-1 activity blocks the expression of IL-10 in LPS-stimulated mouse macrophages (28). IL-10 is an inflammatory inhibitor. Our study detects that the expression of IL-10 in IO group is increased, which may be related to the increased expression of HO-1. However, after the degradation of HO-1-mediated heme, the release of Fe^2+^ shows a pro-oxidative effect and cytotoxicity (29), may aggravate the *in situ* hemolysis and shorten the RBC lifespan in MDS patients.These inflammatory cytokines were decreased significantly after ICT. Regular ICT treatment can significantly reduce the expression of these inflammatory cytokines even in MDS patients with a severe increase of LVIC. The decrease of inflammatory factors may also be one of the reasons why the lifespan of RBC is prolonged.

In conclusion, iron overload is significantly shortens the lifespan of RBC in MDS patients. The shortening of RBC lifespan is not equal to the increase of ineffective hematopoiesis,but iron overload causes the increase of endogenous EPO expression, which leads to the increase of erythroblasts. The increase of ineffective hematopoiesis may be one of the reasons for shortening the lifespan of erythrocytes in MDS patients. The increase of inflammatory cytokines caused by iron overload may damage the erythroblast of MDS combined with morbid hematopoiesis, which is also the reason for shortening the lifespan of erythrocytes.Iron removal therapy can prolong the lifespan of RBC by reducing the production of endogenous EPO and the expression of inflammatory cytokines. Deficiencies of this study: the number of cases in the whole and each subgroup is small, and the influence of erythrocyte transfusion before RBC lifespan testing cannot be completely excluded, so it is best to conduct a large sample of prospective ROT experiments to further confirm.

## Data availability statement

The raw data supporting the conclusions of this article will be made available by the authors, without undue reservation.

## Ethics statement

The studies involving human participants were reviewed and approved by Ethics Committee of Shanghai Sixth People’s Hospital. The patients/participants provided their written informed consent to participate in this study.

## Author contributions

YZ: Experimental design, statistical analysis and thesis writing. YQ: Collection and arrangement of experimental data. L-XS and CX: Assist to collect and arrange experimental data. C-KC: Provide the overall idea and thesis modification. All authors contributed to the article and approved the submitted version.

## Conflict of interest

The authors declare that the research was conducted in the absence of any commercial or financial relationships that could be construed as a potential conflict of interest.

## Publisher’s note

All claims expressed in this article are solely those of the authors and do not necessarily represent those of their affiliated organizations, or those of the publisher, the editors and the reviewers. Any product that may be evaluated in this article, or claim that may be made by its manufacturer, is not guaranteed or endorsed by the publisher.
